# Medical Graduates in the U. S.

**Published:** 1840-06

**Authors:** 


MEDICAL GRADUATES IN THE UNITED STATES IN 1840.

University of Pennsylvania 163; Transylvania University 60; Jefferson Medical College 57; Medical Institute of Louisville 39; Washington University of Baltimore 19; University of Maryland 14; Medical Department of Pennsylvania College 25; Medical College of South Carolina 65.

It has been remarked truly, that the proportion of Students of Medicine to the population of the United States, is greater than exists in any other country. The population of Prussia is thirteen millions, and in 1835 the number of native students there was 699. In France, the same year, with a population of thirty-three millions there were 2, 672 Students of Medicine, many of whom were foreigners. The number in attendance upon all the Medical Schools in the United States, last winter, we have elsewhere stated at about 2,450. With less than half the population of France, we have an equal number of native Students of Medicine, and the fact, we suppose, may thus be accounted for. 1st. The nature of our political institutions favors the multiplication of professional men. Every man who is able educates his sons, and all offices are open alike to all classes of citizens. The professions of law and medicine are the chief roads to distinction, and being free to all are crowded by candidates. 2d. The amount of disease in the United States is proportionably greater than in any of the countries of Europe. This fact, we presume, is well established, and explains the necessity for a more numerous medical corps. 3d. The sparseness of our population greatly increases the labor of physicians, and makes a larger number necessary. After all, probably more engage in the profession than find it profitable, and hence it is that so large a number, after attending one course of lectures, and more after taking their degrees, abandon it for other pursuits. Y.

